# Supplementary material for: The in vivo RNA structurome of the malaria parasite Plasmodium falciparum, a protozoan with an A/U-rich transcriptome
Source: PLoS One. 2022 Sep 1;17(9):e0270863. doi: 10.1371/journal.pone.0270863 (PMC9436142; doi:10.1371/journal.pone.0270863)
Supplement: S1 Raw images — (A) results after probing with DMS (2 concentrations), NAI, vehicle control, i.e. DMSO, or no treatment (UT). DMS and NAI modifications are detected as reverse-transcriptase stops, with very similar patterns at both DMS concentrations. The vehicle control confirms that there is negligible ‘background’, i.e. almost no reverse-transcriptase stops, yielding a near-empty lane similar to the untreated control. (B) full-width gel that appears cropped (as highlighted in red) in Fig 1B, visualised with Fujifilm FLA 9000. (PDF) [file pone.0270863.s001.PDF]

Supplementary figure 1

A

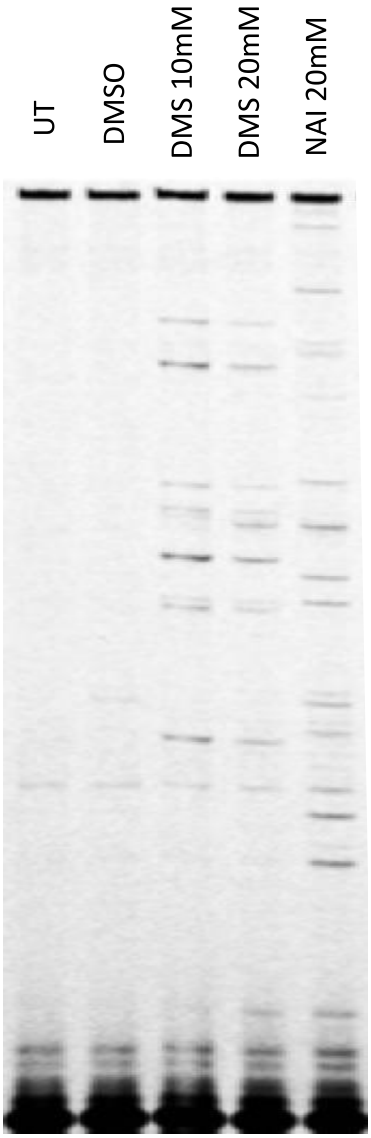

B

Image cropped in Fig 1B is highlighted

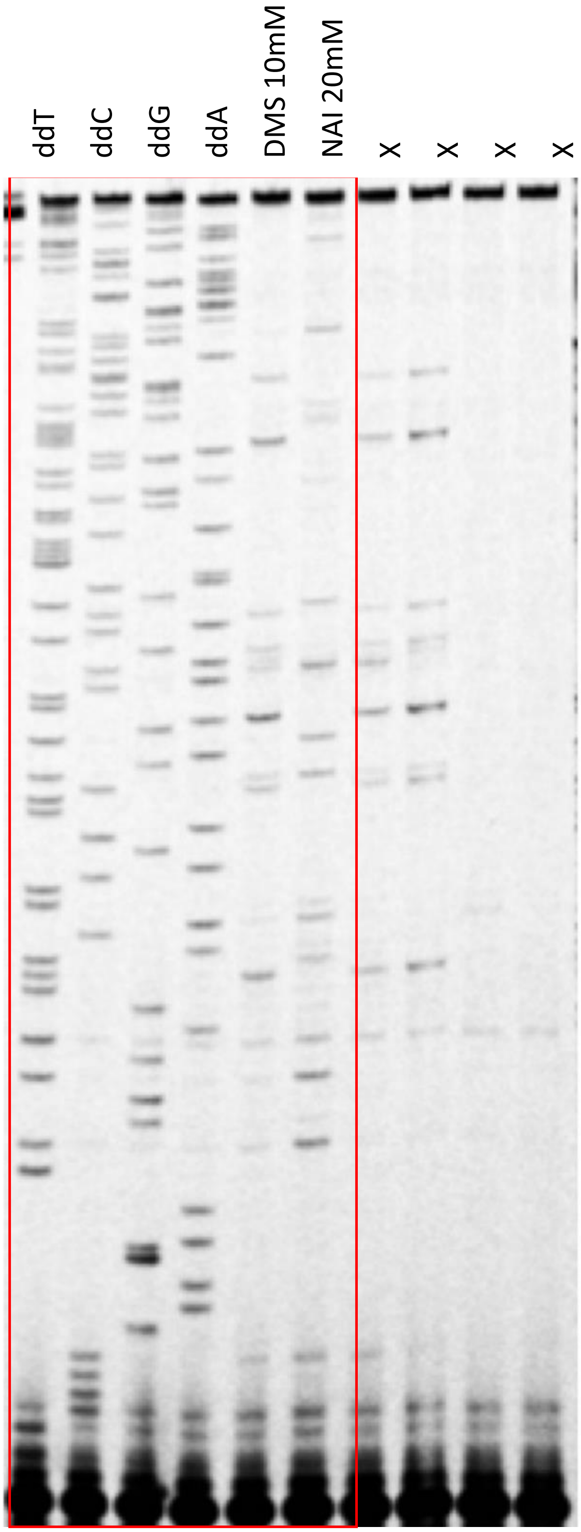

Polyacrylamide gels showing the reverse-transcribed 5.8S rRNA gene of *P. falciparum*.

(A) results after probing with DMS (2 concentrations), NAI, vehicle control, i.e. DMSO, or no treatment (UT). DMS and NAI modifications are detected as reverse-transcriptase stops, with very similar patterns at both DMS concentrations. The vehicle control confirms that there is negligible ‘background’, i.e. almost no reverse-transcriptase stops, yielding a near-empty lane similar to the untreated control.

(B) full-width gel that appears cropped (as highlighted in red) in Figure 1B. Lanes not included in the final figure are marked X.

Method of image capture: Fujifilm FLA 9000. Molecular weight markers: n/a, this is a sequencing gel.
