# Supplementary figures and images for: The in vivo RNA structurome of the malaria parasite Plasmodium falciparum, a protozoan with an A/U-rich transcriptome
Source: PLoS One. 2022 Sep 1;17(9):e0270863. doi: 10.1371/journal.pone.0270863 (PMC9436142; doi:10.1371/journal.pone.0270863)

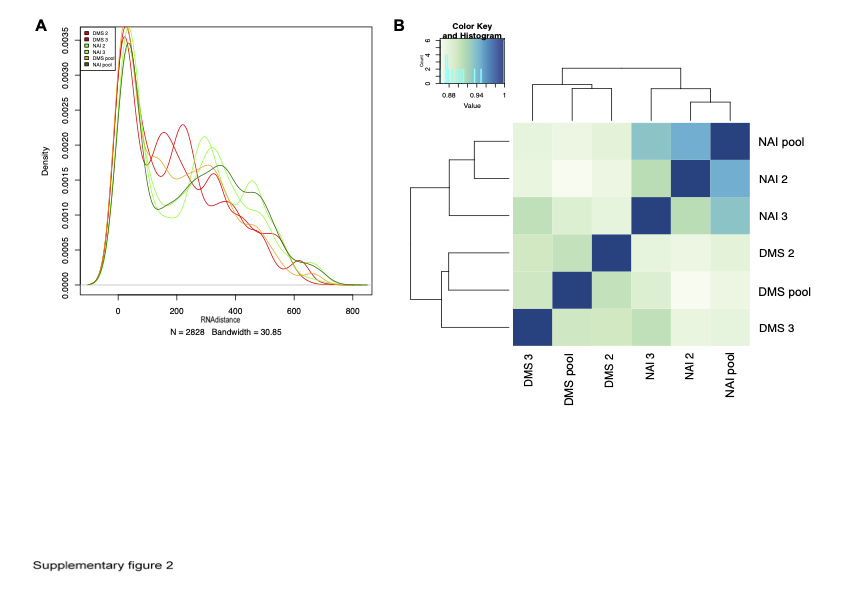

Supplement: S1 Fig — (A) For each of the different treatments (both individual replicates and pooled), the distribution of ‘RNAdistance’ is plotted, i.e. the computed distances between the purely computationally-determined structures and those guided by reactivity data. The y-axis represents the frequency of RNAdistance metrics, which are represented on the x-axis. Replicates of each treatment appear similar, as quantified in (B). (RNAdistance calculated as per: R. Lorenz, S.H. Bernhart, C. Hoener zu Siederdissen, H. Tafer, C. Flamm, P.F. Stadler and I.L. Hofacker (2011), "ViennaRNA Package 2.0", Algorithms for Molecular Biology: 6:26). B) Pearson correlation heat map showing how the RNAdistance-derived distributions in panel A correlate across samples. Two distinct groupings are clearly formed for NAI and DMS. (TIFF) [file pone.0270863.s002.tiff]

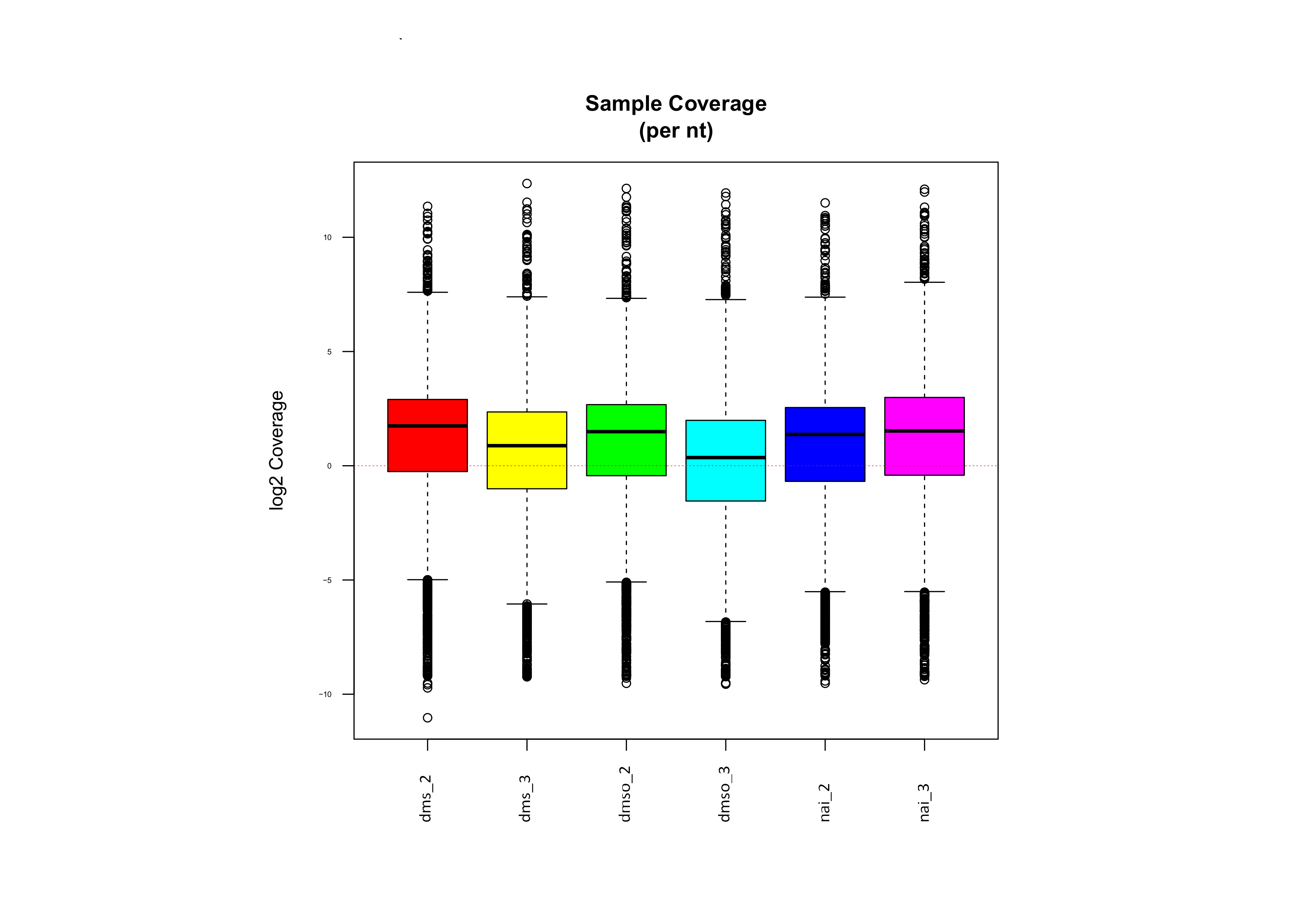

Supplement: S2 Fig — More than 50% of transcripts in every condition met the coverage threshold of >1.0 per nucleotide. (TIF) [file pone.0270863.s003.tif]

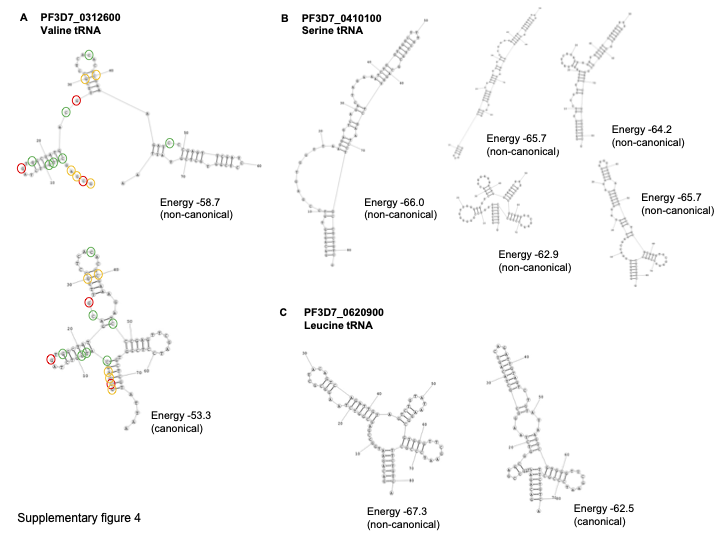

Supplement: S3 Fig — (A) valine tRNA (PF3D7_0312600), (B) serine tRNA (PF3D7_0410100) and (C) leucine (PF3D7_0620900). The structures in (A) and (C) could also be folded into a less-energetically favourable canonical shape: to exemplify this, NAI-reactive bases in (A) are marked on both structures (red, orange, green from highest to lowest relative reactivity). The structure in B could not be folded into a canonical shape, even at 50% less favourable energy, when using the constraints obtained by NAI probing. (TIFF) [file pone.0270863.s004.tiff]

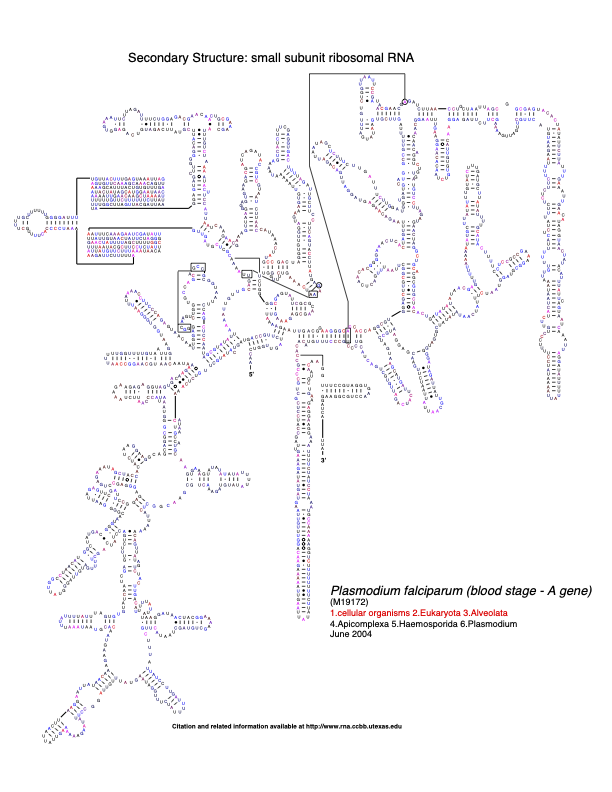

Supplement: S4 Fig — The canonical structure of the P. falciparum 18S rRNA (from https://crw-site.chemistry.gatech.edu/) was compared with base reactivity data for the assembled sequence of the PF3D7_0725600 gene (encoding blood-stage-expressed 18S rRNA). Maximum base reactivities for both the NAI and DMS datasets were mapped: NAI reactivities in the blue channel and DMS reactivities in the red channel, hence dually-reactive bases appear pink. Reactivities were scaled to colour intensity. (TIFF) [file pone.0270863.s005.tiff]
